# Synthetic Bone Grafting in Aseptic Loosening of Acetabular Cup: Good Clinical and Radiological Outcomes in Contained Bone Defects at Medium-Term Follow Up

**DOI:** 10.3390/ijerph17155624

**Published:** 2020-08-04

**Authors:** Paolo Domenico Parchi, Matteo Simonetti, Enrico Bonicoli, Nicola Piolanti, Michelangelo Scaglione

**Affiliations:** 1st Orthopedic Division, Department of Translational Research and new technology in medicine and surgery, University of Pisa, 56124 Pisa, Italy; paolo.parchi@unipi.it (P.D.P.); enrico.bonicoli@gmail.com (E.B.); piolanti.nicola@gmail.com (N.P.); michelangelo.scaglione@gmail.com (M.S.)

**Keywords:** bone grafting, bone substitutes, total hip arthroplasty revision, acetabular cup, hydroxyapatite, osteointegration

## Abstract

Restoring bone loss is one of the major challenges when facing hip revision surgery. To eliminate the risk of disease transmission and antigenicity of allografts and donor-morbidity of autografts, the use of synthetic bioceramics has become popular in the last decade. Our study investigated the effectiveness of impaction bone grafting (IBG) of contained acetabular defects (Paprosky 2 and 3a) using a porous ceramic-based hydroxyapatite bone substitute (Engipore, provided by Finceramica Faenza S.p.A., Faenza, Italy) mixed with a low percentage of autologous bone (obtained from reaming when available). We retrospectively assessed 36 patients who underwent acetabular revision using IBG using a porous ceramic-based hydroxyapatite bone substitute with cementless implants with a mean follow-up of 4.4 years. We evaluated, at regular intervals, patients clinically (using the Hip Harris Score and Oxford Score) and radiologically to evaluate the rate of incorporation of the graft, the presence of radiolucent lines or migrations of the cup. Clinical scores significantly improved (WOMAC improved from 49.7–67.30, and the HSS from 56–89). The rate of implants’ survival was 100% at our medium follow-up (4.4 years). We reported five cases of minor migration of the cup, and radiolucent lines were visible in seven patients at the last-follow up. The graft was well-incorporated in all patients. The results presented in this study suggest the HA bone substitute is an effective and safe bone graft when facing hip revision surgery; thus, longer follow-up studies are required.

## 1. Introduction

With the ageing of the population and a life expectancy increase in the Western world, there will be a greater demand for total hip replacement procedures and, subsequently, of hip revisions.

In the United States, more than 300,000 total hip replacements (THAs) are performed yearly and, according to the National Inpatient Sample of the US, over 250,000 THA revisions were performed between 2009 and 2013 [1]. Given the premises, the revision surgery rate is expected to grow by 137% by the end of 2030 [2].

The main indications for revision surgery are hip instability and aseptic loosening of the implant, accounting for 42% of all revision procedures [3].

The greatest concerns for orthopedic surgeons dealing with a THA revision is how to address bone loss and poor bone regenerative potential to restore the anatomical hip center and improve joint biomechanics.

Impaction bone grafting (IBG) has been described for the treatment of contained defects where a good primary stability could be obtained [4]. Although long-term results with this procedure are mainly related to the surgical technique, good outcomes have been reported in the literature.

IBG of the acetabulum, first performed by Parker et al. in 1975 [5], was made popular by Slooff et al. in 1984 using a cemented acetabular component [6]. More recently, favorable results have been reported when morcellized allografts were used in combination with uncemented cups [7,8,9].

There are multiple viable sources for bone grafting. Autografts are described as the gold standard source to treat bone loss in terms of the properties of osteoconduction, osteoinduction and osteogenesis, but they are rarely used in THA revisions due to high rates of morbidity at harvest sites and their limited availability [10]. Allografts retain osteoconductive properties and may exhibit osteoinductivity potential. For these reasons, although the option presents high costs and there are still concerns about the related risks of disease transmission and antigenicity [11], they could be considered the second-choice option to address bone defects in THA revision procedures.

All the above concerns have led to growing research in developing synthetic bone substitutes for natural bone stock replacement.

Bioactive ceramics are synthetic bone substitutes which have received great attention recently due to their potential in stimulating cell proliferation, differentiation and bone tissue regeneration [12]. Several synthetic ceramics have been tested and used, such as calcium phosphate, tricalcium phosphate, calcium sulphate and hydroxyapatite (HA) [13].

In this scenario, hydroxyapatite (HA), a major component of natural bone, can combine with tissues by chemical bonds to form new bone tissue when implanted [14].

The ceramic-based hydroxyapatite bone graft substitute Engipore (provided by Finceramica Faenza S.p.A., Faenza, Italy) consists of porous hydroxyapatite that allows the product to serve as a scaffold to guide bone regeneration, fostering cell attachment and proliferation and promoting osteointegration [15]. The aim of this retrospective study was to analyze the osteointegrative properties of Engipore bone grafts in revision surgery after primary THA.

## 2. Materials and Methods

This is a retrospective case series of patients who underwent an acetabular revision associated with the use of the bone substitute Engipore between January 2014 and December 2016.

In this period, 105 THA revision procedures were performed: we selected 36 patients whose indication for THA revision was aseptical loosening of the acetabulum after primary THA with a contained bone defect (Paprosky 2 and 3a) treated with cementless implants.

There were 21 women (58.3%) and 15 men (41.7%). The mean age at surgery was 72 (age range: 40–81 years). Patients with an uncontained defect requiring the use of mesh or a cage to reconstruct the acetabulum were excluded from this analysis. It was the first revision in all cases.

We performed a postero-lateral approach to the hip in all patients, obtaining a good exposure of the acetabulum. After implant removal, necrotic and soft tissue surrounding the acetabulum and the bone defect were carefully removed in order to evaluate the severity of bone loss according to Paprosky’s classification (see Table 1) [16].

We prepared the acetabulum with progressive hemispherical reamers and then packed the bone loss with reverse reaming and trial prosthesis using a mixture of Engipore chips mixed with the patient’s blood and autologous bone recovered from reaming when available.

The bone substitute Engipore is a biomimetic and biocompatible porous stoichiometric hydroxyapatite bone substitute comprised of calcium ions, phosphate ions and hydroxyl groups, which is very similar in microstructure and chemical composition to the mineral component of human bones. The trabecular structure, which resembles the mineralized phase of natural bone, is characterized by a 90% porosity rate, allowing physiological fluids absorption, the promotion of cell migration and adhesion for mineral matrix synthesis, thus offering an ideal environment for new bone formation and tissue restoration [17]. The composition, shape and handling properties of this bone substitute make it an ideal bone graft candidate for hip revision surgery. Moreover, this material can be safely mixed with autologous bone. The chips provided by Finceramica used in our study came in the size of 2–4 mm.

**Figure 1:**
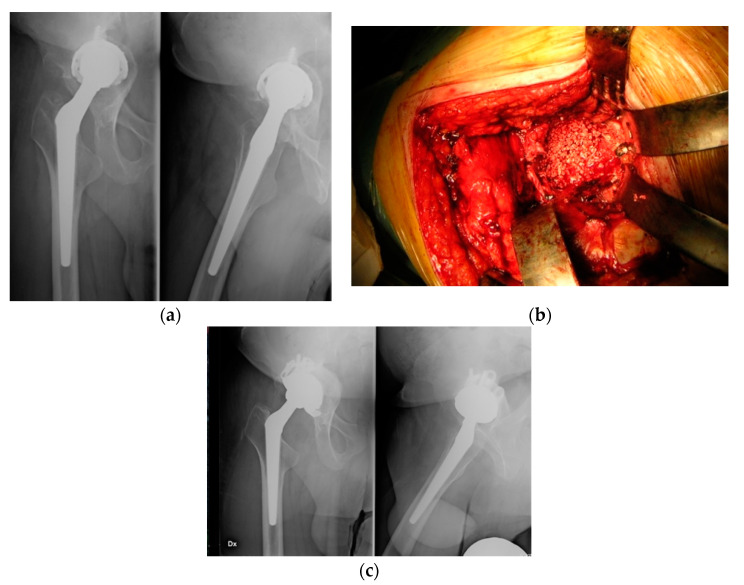
Clinic case: 76 y female with loosening of the acetabular cup (**a**) Pre-operative x-rays, (**b**) Intraoperative pictures show the acetabulum impacted with Engipore chips filling the bone defect (Paprosky 3a), (**c**) Post-operative x-rays at the last follow-up (3.2 years) showing good osteointegration of the implant.

Our case series is comprised of selected patients with contained defects, so it was possible to achieve a primary press-fit stability placing uncemented cups in all cases (Regenerex Revision Shell in 17 cases, Delta TT Revision Cup in 19 cases), 1–2 mm larger than the last trial used. Regardless of the primary stability of the implant, a median of 4 screws (2–6) was used to fix the shell (Figure 1). The post-operative rehabilitation protocol included: mobilization of the hip, avoiding luxation movements, and partial weight bearing was given for 6 weeks and then gradually progressed to full weight bearing and was performed by all the patients. Clinical and radiographical evaluations of all patients were performed pre-operatively and post-operatively at regular intervals (1, 3, 6 months and yearly after). The radiological follow-up included anterior-posterior X-rays of the pelvis and antero-posterior and lateral X-rays of the hip. The clinical evaluation was conducted using the Harris Hip Score (HHS) [18] and the West Ontario and McMaster Universities Osteoarthritis Index (WOMAC) [19]. We eventually reached by phone patients (or their relatives) who did not fully accomplish follow-up visits to assess whether they had underwent further surgery. Radiographic analysis was carried out by two senior surgeons (N.P. and M.S.), assessing graft incorporation, bone resorption or migration of the implant. We used the De Lee and Charnley classification [20] to assess lines of radiolucency around the acetabular component. A radiolucent line wider than 2 mm was considered significant. We considered the surgery to have failed if the cup had migrated 3 mm or more or if revision was necessary. The grade of heterotopic ossification was evaluated according to Brooker et al. [21]. The mean follow-up was 4.4 years (minimum 3.1—maximum 5.8 years). Given the small sample size, statistical testing of correlations was not determined to be useful.

### Ethical Statement

This is retrospective study which was approved by internal revision board and for these reason needs only a tacital approval of the ethical committee—ethical approval was not needed for this study.

## 3. Results

Thirty-six patients met the inclusion and exclusion criteria defined for our retrospective case analysis. Five patients died of disease not related to the surgical procedures. Among the remaining 31 patients, 25 patients returned a complete questionnaire; meanwhile, a full radiological follow-up was available in 28 cases.

We recorded a significant improvement in clinical function: the WOMAC score changed from 49.7 pre-operatively to 67.3 post-operatively, and the HHS changed from 56.1 pre-operatively to 89.4 post-operatively. At the final follow-up, most of the patients (67%) stated they would undergo surgery again. Data about survivorship of the implant were available for all patients. There were no cases of acetabular revision in the selected patients, so the rate of survivorship of the implant at our medium follow-up was 100%. We reported five cases of acetabular migrations wider than 3 mm (range: 3–6 mm), but surgery was not necessary because four patients reported no functional impairment and one patient had functional impairment but refused further surgery. All migrations occurred within one year after surgery and then remained stable during the follow-up and were not associated with significant worsening of the clinical scores. According to De Lee and Charnley et al. [20], we found at the initial follow-up two patients with radiolucent lines bigger than 1 mm in zone 1, three patients in zone 2, one patient in zone 3, two patients both in zone 1 and 2 and one patient in all three zones (see Table 2). No significant association between the presence of radiolucent lines and clinical outcome was noticed.

**Figure 2:**
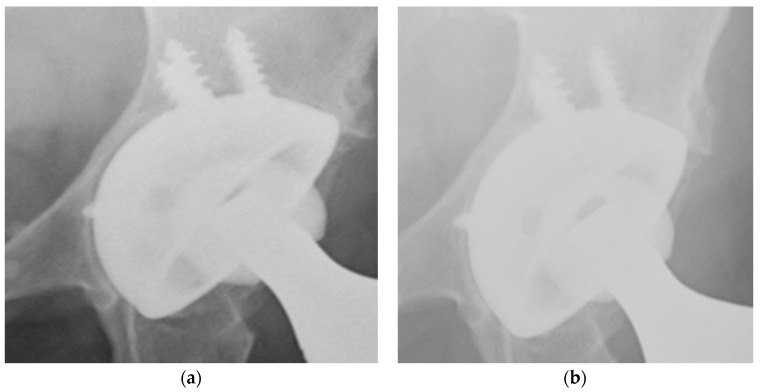
X-rays show good osteointegration of the implant and formation in a 72-year-old woman treated with cup revision for a Paprosky 3a. (**a**) One month follow-up, presence of radiolucent lines, (**b**) One year follow-up, shows filling of the radiolucent line with the formation of new bone.

In the last X-ray evaluation, radiolucent lines remained still visible and no signs of progression were detectable, except for two cases, in which radiolucent lines were filled by, presumably, the formation of new bone (see Figure 2).

Mild heterotopic ossification (type 1–2 according to the Brooker classification) was found in three patients. Early complications (during the in-patient stay) occurred in five of the 31 patients. These included a superficial wound infection in two patients, treated successfully with antibiotic therapy; one case of deep infection treated successfully with DAIR (debridement, antibiotics and implant retention); one case of deep-vein thrombosis and one case of postoperative early dislocation, which was successfully treated with close reduction. One patient sustained a periprosthetic fracture of the femur (type B1 according to the Vancouver Classification) after falling and was treated successfully with osteosynthesis with a plate.

## 4. Discussion

There is a great debate in the literature regarding clinical and radiological outcomes of hip revision surgery using synthetic bone substitutes. Kurien et al. [22], in a systematic review about the evidence of the use of bone graft substitutes, stated that there are few synthetic graft substitutes with level I evidence. A certain number of papers have reported good results on the use of bone substitutes in hip revision surgery, but they were all a heterogenous case series mix with different kinds of implants, wide bone loss severity and type of bone substitutes used. Our case series, even if small, has an asset of being a homogeneous court of patients characterized by an isolated acetabular contained defect treated with hydroxyapatite (mixed with autologous bone and blood obtained from reaming when available) and with cementless implants. IBG is recognized as an efficient method to treat contained acetabular defects [16,23], even if the procedure is described as successful for selected cases of the Paprosky 3 type [24]. A porous, cementless coated socket would lead to bony ingrowth and osteointegration, providing a stable and solid fixation [7]. Few authors have reported the results of the use of an isolated bone graft substitute, not augmented with allografts or autografts. Oonishi et al. [25] documented the use of hydroxyapatite granules to fill massive bone loss in 40 patients using cemented sockets, obtaining good clinical and radiological results in a 4–10 year follow-up. They reported three cup migrations associated with mild clinical impairment. Good osteointegration to native bone was observed in all 40 patients. Schwartz et al. [26] used a biphasic phosphor-calcium bone substitute to face severe acetabular bone loss using both jumbo cups and a screwed support ring. At a mean 10 year follow-up, they reported no cases of migration of the cup in living patients and good bone osteointegration. Coralline hydroxyapatite was used by Wasielewski et al. [27] in complex acetabular revision surgery, reporting one case of failure. No resorption of the graft was noticed and all cases showed good osteointegration. Our clinical results encourage the choice of synthetic bone substitutes, rather than a metallic augment, when facing THA revision. Patients were eventually satisfied in terms of pain relief and functional recovery, and most of them stated they would undergo surgery again. The radiographical results are quite in contrast to the clinical outcomes: we registered five cases of acetabular migration, of which one was considered a frank radiological failure but not associated with worsening of clinical scores. The incidence of radiolucent lines we reported around the acetabular shells was concerning, but similar to other studies in the literature [27,28]. Although these radiolucent lines showed up early after surgery, they did not become wider with the progress of time; thus, we agree with Schmalzried et al. [29], and we consider that these radiolucent lines could represent a predictive factor of future aseptic loosening, so patients need to be kept monitored. The rate of graft incorporation in our series was satisfactory, similar to others reported [25] or even higher compared to other series in the literature [30]. We reported only one patient with graft incorporation failure in all three zones, but the case was not associated with any clinical impairment. Synthetic bioceramics such as Engipore offer several advantages over autografts and allografts in terms of safety [10]. The synthetic fabrication of Engipore makes the product free of any risk of disease transmission or immunoreaction, and the availability of a wide range of shapes and formats makes it a valid option for different surgical applications. Moreover, its availability off-the-shelf eliminates the donor-morbidity of autograft harvests and reliance on bone banks for allograft supply.

Based on this clinical experience, as a general comment, the identification of the most appropriate surgery, together with a proper application and packing of the chips in the surgical implantation phase, are key aspects for a successful outcome.

## 5. Conclusions

The clinical and radiological results presented in our study suggest that the bioceramic bone substitute Engipore can be used as an ideal bone substitute in THA acetabular revision surgery, even if mixed with a low percentage of autologous bone. The product is safe, with no risk of disease transmission or an antigenic response. It led to a low rate of failure and a satisfactory grade of osteointegration. The results presented in the study must be interpreted with caution due to the low number of patients enrolled and the retrospective design of the study and follow-up period. Prospective, randomized controlled clinical studies would be beneficial to confirm its safety and efficacy at a longer follow-up.

## Figures and Tables

**Table 1:** Paprosky’s classification of acetabular bone loss.

Type	Description	Patients	Mean Age
1	Minimal deformity, intact rim	-	-
2a	Superior bone lysis with intact superior rim	10	69 years
2b	Absent superior rim, superolateral migration	8	70 years
2c	Localized destruction of medial wall	11	73 years
3a	Bone loss from 10 am to 2 pm around rim with 30–60% of the supporting bone stock destroyed. There is superolateral cup migration	7	76 years
3b	Bone loss from 9 am to 5 pm around rim with up to 60% of the supporting bone stock destroyed. There is superomedial cup migration	-	-
4	Pelvic discontinuity	-	-

**Table 2:** Clinical and radiological results at the mean follow-up (4.4 years).

Clinical and Radiological Results	No.
Mean pre-operative WS	49.7 (39–57)
Mean post-operative WS	67.3 (53–81)
Mean pre-operative HHS	56.1 (41–75)
Mean post-operative HHS	89.4 (62–91)
Radiolucent lines at the last follow-up	7 pz
Migration >3 mm (radiological failure)	5
Clinical failure	1
Eterotopic ossifications	3

HHS, Harris Hip Score; WS, WOMAC Score.
